# The function of timbre in the perception of affective intentions: Effect of enculturation in different musical traditions

**DOI:** 10.1177/10298649241237775

**Published:** 2024-03-18

**Authors:** Lena Heng, Stephen McAdams

**Affiliations:** Schulich School of Music, McGill University, Montreal, QC, Canada

**Keywords:** timbre, musical affect, acoustic features, cross-cultural differences, music perception

## Abstract

Timbre has been identified as a potential component in the communication of affect in music. Although its function as a carrier of perceptually useful information about sound source mechanics has been established, less is understood about whether and how it functions as a carrier of information for communicating affect in music. To investigate these issues, listeners trained in Chinese and Western musical traditions were presented with Phrases, Measures, and Notes of recorded excerpts interpreted with a variety of affective intentions by performers on instruments from the two cultures. Results showed greater accuracy and more extreme responses in Chinese musician listeners and lowest accuracy in nonmusicians suggesting that musical training plays a role in listeners’ decoding of affective intention. Responses were more differentiated and more accurate with more musical information. Excerpts were also analyzed to determine acoustic features that are correlated with timbre characteristics. Temporal, spectral, and spectrotemporal attributes were consistently used in judging affective intent in music, suggesting purposeful use of these properties by listeners. Comparison between listeners’ use of acoustic features reveals a greater number of shared features between Western musicians and nonmusicians compared to Chinese musicians for valence, although the three groups shared more features for arousal. How timbre is utilized in musical communication appears to be different across musical traditions, and valence responses seem to be more culture-specific and arousal responses more similar across cultures.

Communicative behavior is shared by conscious living beings (Dance, 1970). The triadic relationship between an object or idea, how this object/idea is referred to, and the perceiver of this object/idea has been widely acknowledged (e.g., Mead, 1934; Pierce, 2014). Others have brought this concept of the triadic relationship in communication into the field of communication in music (e.g., Thoresen, 2015). Even so, there are still lacunae in the study of signification in musical communication. As Nattiez (1990) argues, objects and ideas are not signified only by language. Music also has the potential for signification. However, the ways in which music can signify meanings might be different from signification in language.

Musical meanings vary widely across cultures (Cross, 2009). In musical cultures with a tradition of codifying musical works as repeatable entities (Western classical music being one, Chinese music being another), we need to consider performers’ and composers’ expression, and listeners’ recognition of intentions in the music when discussing musical meanings. In the present article, we will define musical meaning as information that is carried by music. On the one hand, the principles of auditory perception enable a listener to parse and process elaborate and abstract sonic information and thus allow for the communication of complex information. On the other hand, Tomlinson (1984) reminds musicologists of the importance of interpreting the cultural context within which the particular form of music arises. In other words, musical meaning has to be inferred using “psychological processes ingrained as habits in the perceptions, disposition, and responses of those who have learned through practice and experience to understand a particular style,” and these constants between styles reveal to us how “the mind, operating within the context of culturally established norms, selects and organizes the stimuli that are presented to it” (Meyer, 1994, p. 1). Meyer uses the term “cultural noise” to refer to “disparities that may exist between the habit responses required by the musical style and those which a given individual actually possesses” (p. 16). As a consequence, listeners with different musical backgrounds and experiences may interpret the same piece of music in different ways. Understanding musical meanings has to take into account the poietic source that derived from a process of creation, as well as the psychological processes and cultural background of listeners (the esthesic process) in reconstructing this “message” (Nattiez, 1990, p. 67).

Although Juslin and Timmers (2010) primarily discuss affective intentions in music, their definition of affect communication is applicable to communication in the more general sense. To capture the characteristics of the communicative process in music, they conceptualize it in terms of a variant of Brunswik’s (1956) lens model whereby the intentions of the performer(s) are expressed by means of a set of cues that are probabilistic and partly redundant, and these are then interpreted by the listener.

Affective intention in music, as an umbrella term covering all aspects of evaluative states with regard to musical communication, is an important and salient notion that may be considered an aspect of musical meaning. Communication of affective intentions involves “both a performer’s intention to express a specific emotion and recognition of this emotion by a listener” (Juslin & Timmers, 2010, p. 455).

Psychologists hold differing views as to which types of models best describe emotions and affect. Those used and cited most often range from Ekman’s (1992) model of universal basic emotions, Shaver et al.’s (1987) model of emotion prototypes, Russell’s (1980) two-dimensional circumplex model of affect (activation-pleasantness, often expressed as arousal-valence in music research), and Schimmack and Grob’s (2000) three-dimensional model of core affect (pleasure-energy-tension). Experimental studies of music, emotions, and affect have tested these models. Eerola and Vuoskoski (2011) compared the discrete and dimensional models and found that Russell’s (1980) circumplex model is sufficient to explain listeners’ judgments of perceived affect in music. Cespedes-Guevara and Eerola (2018) argue for a constructionist approach to the perception of emotion in music saying that “it may be more parsimonious to assume that music communicates fluctuations of affect which can be mapped onto many possible meanings via associative mechanisms” (p. 14). Even when listeners ascribe categorical or prototypical emotions to the music they hear, these emotions are varied and nuanced. It can therefore be assumed that regardless of whether or not listeners place the music they hear into specific emotion categories or otherwise, they have an understanding of its affective intentions as predicted by a dimensional model, although the number of dimensions may vary. In the present study, we did not aim to compare discrete and dimensional models. On the basis of the evidence for the two-dimensional model of valence and arousal, and for the sake of parsimony, we accepted this explanation of listeners’ understanding of musical meaning as a tool for measuring reception of affective intentions.

Music can communicate and elicit affect and emotions in many ways (Juslin & Laukka, 2004). Various dimensions of musical sound carry important information for the communication of affective intentions. These include the structure of the composition, elements usually “represented by designations in the conventional musical notation, such as tempo markings, dynamic markings, pitch intervals, mode, melody, rhythm, harmony, and various formal properties” (Gabrielsson & Lindström, 2010, pp. 367–368), and elements that are realized in performance (Juslin & Timmers, 2010, p. 458). McAdams (1989) discusses the constraints of these dimensions on their potential for bearing form. Well-known bearers of the form include pitch and duration, and the contributions of these to the interpretation of musical meanings have often been studied. Timbre also has a strong potential to bear form, although there has been less extensive work on this musical dimension (but see McAdams, 2019b).

Timbre has been shown to carry perceptually useful information about sound source mechanics. However, even though it has also been identified by music perception researchers as a component of the communication of affect in music (e.g., Eerola et al., 2012; Eerola & Vuoskoski, 2011; McAdams et al., 2017), there is still a lot to discover about how it functions as a carrier of information. The timbral characteristics of an instrument have been found to influence listeners’ perception of emotion (Hailstone et al., 2009). Studies have also shown the influence of timbre manipulation in performance on perceived emotion (Juslin & Timmers, 2010). McAdams (2019a) has recently defined timbre as a “complex auditory attribute, or as a set of attributes, of a perceptually fused sound event in addition to those of pitch, loudness, perceived duration, and spatial position. . .[and] is also a perceptual property, not a physical one” (p. 23). Even though timbre is a psychophysical attribute, and it is the perception of a sound that defines timbre, we must not underestimate the importance of the physical acoustic properties that give rise to timbre perception. As surface acoustic properties carry important information for perception, a systematic approach to sound analysis that is oriented toward human perception (Peeters et al., 2011) and its relation to the communication of affective intentions in music will greatly aid the understanding of timbre’s function in musical communication.

With regard to the attribution of affective intentions to musical sounds, Scherer and Oshinsky (1977) systematically manipulated certain acoustic parameters to study listeners’ ratings of both discrete emotions and dimensional affective intentions. Other researchers since then have also found consistent mappings of acoustic cues to affective communication (e.g., Bowman & Yamauchi, 2016; Eerola et al., 2012; McAdams et al., 2017; Schimmack & Grob, 2000; Soden et al., 2019). There is much overlap in the acoustic dimensions that seem to be involved in carrying these affective intentions, suggesting that affective content is not just related to a single acoustic dimension but is also communicated through the complex combinations and interactions of several acoustic parameters. Juslin (2000) and Scherer and colleagues (2011) applied a modified lens model to the process of expression and perception. In their model, specificities or uniformity in cues do not have to be present in decoding. In other words, listeners may utilize many different strategies or attend to different cues in flexible ways and still be successful at decoding intentions.

Thompson and Balkwill (2010) argue that musical experiences are constrained in important ways in several aspects, including the environment, the structure of the auditory system, and the nature of perception and cognition. Commonalities allow for the cross-cultural understanding of affective intention in music, whereas culture-specific understanding is built upon these constraints through processes of enculturation. Their cue-redundancy model explains that listeners across different cultures are able to appreciate the affective qualities of unfamiliar music by attending to these commonalities, whereas listeners who are familiar with a musical style should find it easier to decode emotional meaning in that music because they can draw from both culture-specific as well as common psychophysical cues. Balkwill and Thompson (1999) investigated the perception of emotion in listeners from different cultural backgrounds by asking participants familiar with the Western tonal system to listen to Hindustani music and identify the dominant emotion present in each piece. They found that their participants were sensitive to emotions in music from an unfamiliar tonal system, and that common psychophysical cues provided sufficient information for decoding when culture-specific cues were unavailable. Thompson and colleagues (2022) also discuss the roles of structure, self, and source appraising in making sense of musical events, which carry with them their contextual framework. Thus, listeners’ cultural backgrounds and experiences play an important role in their comprehension of music.

Studies have revealed some differences between listeners from different cultures—Chinese and Western—in the multidimensional space obtained from rating dissimilarities of instrument sounds (e.g., Zhang & Xie, 2017). Although these differences might have been due to the different sets of instruments used (Chinese vs. Western instruments), the different dimensions obtained from the multidimensional scaling could also imply a focus on different aspects of a sound by different groups of listeners (McAdams et al., 1995). Wang and colleagues (2021) also found that both cultural and musical backgrounds influence emotion perception in music. Egermann and colleagues (2015) found that listeners from different cultures (Pygmy and North American) had more similar arousal responses when presented with Western and Pygmy music but differed more in their valence responses. They suggested that “music-induced arousal responses appeared to be based on rather universal, culturally independent response mechanisms” (p. 8). Less research has been done on listeners with different musical cultures but from the same geographical location. With participants from the same geographical location, differences in linguistic and socio-cultural factors can be better controlled, and effects due to musical training more clearly demonstrated. In our study, we recruited three groups of participants from Singapore with different musical backgrounds. The first and second groups were trained in either Chinese or Western musical traditions (musicians: CHM or WM). The third group was nonmusicians (NM).

## Research goals and hypotheses

The goal of this study was to extend previous research on affect perception in music listening. We included factors of listener group, length of musical context, instrument category, and instrument culture. We focused on the influences that training in different musical cultures has on listeners’ perception of affective intentions in music and their relation to the affect intended by performers on Western and Chinese instruments, who were asked to express a melody in the four quadrants of the arousal-valence space. We also examined the amount of musical information available to decode the intention. Furthermore, we explored the ways in which listeners with different cultures of musical training utilize acoustic cues in their perception of affective intentions. We proposed four hypotheses.

H1. There would be an effect of musical context on decoding accuracy such that the percentage of accurate responses (a match between performer intention and listener response) is higher for Phrases than for Measures, and for Measures than for Notes. This hypothesis was tested by the main effect of context.
H2. There would be an effect of musical training on decoding accuracy such that the percentage of accurate responses is higher for musicians than NM, regardless of the musical tradition of the instrument used for the stimuli, because they have cues available to them from their formal learning and experience in music that are not available to NM. This hypothesis was tested by the main effect of the listener group.
H3. There would be effects of instrument culture of musical training such that CHM would have higher decoding accuracy for stimuli played on Chinese instruments than WM or NM because they have the largest number of culture-specific cues available to them. Similarly, WM would have higher decoding accuracy for stimuli on Western instruments than CHM and NM. This hypothesis was tested by the listener group × instrument culture interaction.
H4. There would be more overlap between the acoustic cues used by CHM and WM for decoding affective intentions than between the cues used by musicians and NM. This hypothesis was tested by a comparison of the number of similar acoustic features loading onto the principal components of a partial least-squares regression (PLSR) of acoustic features onto valence and arousal ratings for each listener group.

We also wanted to explore three additional points for which we did not have specific hypotheses to be tested.

E1. Whether specific affective intentions are expressed more effectively by performers on certain categories of instruments for different listener groups. This point would be explored through interaction effects between the listener group, affective intention, and instrument category.
E2. Whether there are differences between the three groups of listeners’ ratings of valence and arousal for each affective intention. This point would be explored through the listener group × affective intention interaction with accuracy measures, as well as with a MANOVA assessing the listeners’ direct ratings of arousal and valence with respect to the intended affective intentions in the stimuli.
E3. The extent to which acoustic cues related to timbre contribute to listeners’ perceptions of affective intentions. This point would be explored by examining the loadings of acoustic features on each principal component of the PLSR.

## Method

### Experimental design

Two dependent variables were recorded concurrently in the listening experiment: arousal and valence ratings in a two-dimensional interface. The ratings were used directly in some analyses and were recoded in terms of their accuracy with respect to the performers’ intentions in other analyses. We used a mixed-factorial design with one between-subjects and four within-subject independent variables. The between-subject variable had three levels: musicians trained in the Chinese music tradition (CHM), musicians trained in the Western classical music tradition (WM), and nonmusicians (NM). The four within-subject variables were 1) performers’ affective intention (four levels: low-arousal negative valence [L−], low-arousal positive valence [L+], high arousal negative valence [H−], and high arousal positive valence [H+]; 2) instrument culture (two levels: Chinese and Western); 3) instrument category (three levels: aerophones, bowed chordophones, and plucked chordophones); and 4) musical context (three levels: Note, Measure, and Phrase).

### Participants

All the participants were recruited from Singapore to reduce the effects of socio-cultural and linguistic differences. There were 90 participants in total (51 female) aged 16 to 62 years (*M* = 24, *SD* = 6.88). They were divided into CHM, WM (musicians), and NM. Musicians had a minimum of five years of training. NM had had less than a year of formal training in any type of music. However, they had all been exposed to both Chinese and Western art music since these are ubiquitous in Singapore. There were 30 CHM, 20 female, aged 16 to 39 (*M* = 26.1, *SD* = 5.73) with a mean of 12 years of formal training (*SD* = 2.98); 30 WM, 19 female, aged 17 to 62 (*M* = 22.67, *SD* = 8.22) with a mean of 12.13 years of formal training (*SD* = 7.40); and 30 NM, 14 female, aged 19 to 41 (*M* *=* 23.23, *SD* = 6.15), with a mean of 0.2 (*SD* = 0.41) years of formal training. There was no significant difference between the ages of the three groups of participants, *F*(2, 87) = 2.289, *p* = .107. There was also no significant difference between the years of training of the CHM and WM groups, *t*(58) = –0.09, *p* = .93. None of the WM group had had any training in Chinese music. Even though four CHM participants had had formal training in Western music for between one and three years, all CHM participants identified themselves as more proficient in Chinese than Western music.

All participants met the required hearing threshold of 20 dB HL on a pure-tone audiometric test with octave-spaced frequencies from 125 Hz to 8 kHz (International Organization for Standardization, 2004; Martin & Champlin, 2000). They signed a written consent form and were compensated for their participation. This study was certified for ethical compliance by the Research Ethics Board II of McGill University.

### Stimuli

The music we used in the experiment was a melody excerpted from an anthology of cue sheets for silent film compiled by George West (1920). We only used one excerpt, to keep the procedure short. We chose it because we assumed that all three groups of participants would have been exposed to the same extent to Hollywood movies, and silent films in particular, so that no group would be biased by its genre or style, that it could carry affective intentions effectively, but that these could be interpreted in different ways. The modality of the excerpt was ambiguous; it begins in the major, goes into the minor, and returns to the major mode.

The music was recorded by six professional musicians (musicians who teach and perform as their regular work) on Chinese and Western instruments consisting of two aerophones (dizi and flute), two bowed chordophones (erhu and violin), and two plucked chordophones (pipa and guitar). We asked them to perform the excerpt at the notated octave. We explained the two-dimensional model of valence and arousal (Russell, 1980) to the musicians and asked them to perform the music in such a way as to convey five different affective intentions: L−, H−, H+, L+, and neutral (N). The assumption was that performers would target extremes as much as possible in their performances, although it was to be expected that not all notes would carry a particular affective intention to the same extent, because this also depends on the performer, the instrument, and the position of each note in a phrase. We recorded the performances in a soundproof room, using a Zoom H4n Handy Recorder (Zoom Corporation, Tokyo, Japan) placed 1 m from the instrument, and adjusted the input level for each instrument. The musicians’ performances of the excerpt, interpreted with different affective intentions, lasted between 11 and 45 s. We subsequently normalized each recorded excerpt so that their peak amplitudes were −1 dBFS.

**Figure 1.**
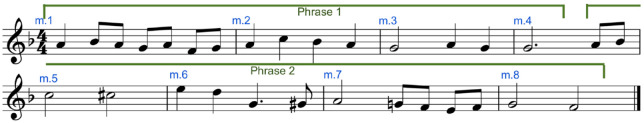
Musical excerpt used with Measures labeled as m.1 to m.8 and Phrases labelled as Phrase 1 and Phrase 2.

We used Audacity® version 2.3.0 (2018) to identify the onsets of 30 individual notes, their durations lasting until the onset of the next note, and extracted them from the recorded stimuli. We extracted eight measures and two phrases in the same way. Each measure or phrase lasted from the onset of its first note to the first note of the next measure or phrase. The whole excerpt, and its division into measures and phrases, is shown in Figure 1. The stimuli were stored on a Macintosh laptop running OSX (Apple Computers, Inc., Cupertino, CA).

We used the revised Timbre Toolbox (Kazazis et al., 2021) implemented in the MATLAB environment to carry out the acoustic analysis. We performed all statistical analyses in *R* (Version 4.2.0; R Foundation for Statistical Computing, Vienna, Austria; R Core Team, 2022).

### Procedure

Each participant participated in two sessions, each lasting about 45 min. The sessions were conducted at least a week apart to reduce potential memory effects, as the stimuli used in the two experiments were obtained from the same recordings. Participants were randomly assigned to undertake either Session 1 (Note) or Session 2 (Measure and Phrase) first.

Participants were seated individually in a quiet room and listened to the stimuli using Sennheiser HD 280 Pro headphones (Sennheiser Electronic GmbH, Wedemark, Germany), with sound levels set to a comfortable listening level for all participants. They were instructed not to adjust the level of the sound during the experiment. The PsiExp computer environment (Smith, 1995) was used to deliver the experiment and collect data. Participants rated the performer’s affective intention by positioning a cursor on a computer screen showing a two-dimensional interface with valence on the *x*-axis and arousal on the *y*-axis. This interface had been found by Schubert (1999) to have good semantic resolution, be intuitive to use, and exhibit high reliability and validity. Before participants started the experiment, the researcher provided verbal instructions and asked questions about valence, arousal, and affect, to ensure that participants understood what was required. Participants then undertook four practice trials to familiarize themselves with the interface and procedure. Valence and arousal ratings were coded with a resolution of .01 and ranged from −1 to +1. In Session 1, participants rated the individual notes, with randomized order of trials. In Session 2, they rated measures and phrases with the Measure and Phrase trials randomized together.

### Analyses

First, we carried out an ANOVA on listeners’ decoding accuracy based on their agreement as to the affective quadrant of the expressed intention. Even though there might be arguments for exploring gradations in listeners’ decoding accuracy, for the purposes of the present study, we determined accurate decoding by whether a participant placed the cursor in the quadrant representing the performer’s affective intention. When a participant positioned their cursor at a position in a particular quadrant of the valence-arousal space, they were assumed to perceive the stimulus to convey that affective intention, even if the position of the cursor was close to the center of the display (0). During the instruction phase, they were also reminded that they should position the cursor on 0 only if they felt that the stimulus expressed neither positive nor negative valence and produced neither high nor low arousal. Listeners’ responses were individually coded into the corresponding emotion-space quadrant (L−, H−, H+, or L+) and responses in the same affective intention quadrant as the one intended by the performer were coded as correct. The same process was carried out for the responses on single Notes, Measures, and Phrases. The percentage of correct responses for each affective intention was then computed for each participant. Due to violations of homoscedasticity assumptions, we performed the aligned rank transform procedure (Wobbrock et al., 2011) on the data before applying a five-way mixed-model ANOVA on the transformed data. We then carried out post hoc comparisons based on a linear model aligned and ranked on the factors (Elkin et al., 2021). The Bonferroni-Holm method was used to adjust the *p* values to ensure that Type I errors are not inflated with the multiple comparisons. Corrected *p* values are reported.

To observe which acoustic features reveal the ways in which timbre cues influence the perception of affective intentions, a PLSR was conducted. Several relevant acoustic features (also referred to as audio descriptors) computed on the stimuli were used as predictors of affect ratings on the arousal and valence scales.

## Results

First, we report listeners’ accuracy in perceiving performers’ affective intentions based on a five-way mixed-model ANOVA followed by post hoc comparisons. Next, we report listeners’ direct ratings of valence and arousal in each of the affective intention conditions based on a two-way mixed multivariate analysis of variance (MANOVA). Finally, we report the results of the PLSR exploring the relationship between acoustic features and listeners’ valence and arousal ratings.

### Length of musical context

A main effect of context with post hoc comparisons showing Phrase > Measure > Note would support the first hypothesis (H1) that increasing musical context provides more information for listeners to accurately decode affective intentions expressed in the performed stimuli. The five-way interaction was significant, *F*(24, 6177) = 3.78, *p* < .001, *η_p_*^2^ = .01, albeit with a very small effect size. The main effect of context was significant, *F*(2, 6177) = 7145.76, *p* < .001, *η_p_*^2^ = .70. Post hoc comparisons showed that accuracy was significantly higher in the Phrase than the Measure context, *t*(6177) = 60.17, *p* < .001, and in the Measure than the Note context, *t*(6177) = 59.37, *p* < .001.

### Differences among listener groups

**Figure 2.**
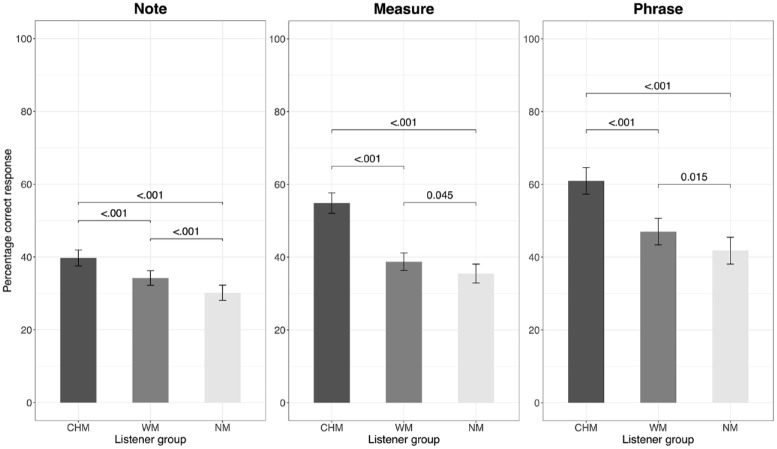
Percentage accurate responses for each listener group with standard error of the mean.

We performed separate four-way ANOVAs for each level of context (Note, Measure, Phrase) to examine further the effects of listener group, affective intentions, instrument culture, and instrument type. A main effect of listener group with post hoc comparisons showing CHM/WM > NM would support the second hypothesis (H2) that musicians perceive performers’ affective intentions more accurately than nonmusicians. Supplementary Tables S1a to S1c show the results of the aligned rank transformed ANOVA separately for accuracy in the three levels of context. There was a significant main effect of listener group in all three contexts: *F*(2, 87) = 62.24, *p* < .001, *η_p_*^2^ = .59 for Note; *F*(2, 87) = 82.64, *p* < .001, *η_p_*^2^ = .66 for Measure; and *F*(2, 87) = 50.45, *p* < .001, *η_p_*^2^ = .54 for Phrase. Figure 2 plots the percentage of correct responses given by each listener group in the three contexts, with post hoc comparisons between listener groups. CHM performed more accurately than both WM and NM, and WM was significantly more accurate than NM in all three context levels. This result confirms our second hypothesis (H2) that musicians decode performers’ intentions more accurately than nonmusicians but additionally reveals that CHM decode intentions better than WM.

### Cultural specificity of musical training

Separate four-way interactions for each context revealed small effect sizes. This interaction was significant for the Measure context, *F*(12, 2001) = 8.15, *p* < .001, *η_p_*^2^ = 0.047, and the Phrase context, *F*(12, 2001) = 3.06, *p* < .001, *η_p_*^2^ = 0.018 but not the Note context. The main effect of instrument culture showed that stimuli performed on Chinese instruments elicited a significantly greater number of accurate responses as compared to those performed on Western instruments in the Note context, but no differences were observed in the Measure or Phrase contexts. An interaction between listener group and instrument culture would support the third hypothesis (H3) that participants would be more accurate in perceiving the affective intention of a stimulus performed by an instrument from the musical culture with which they were more familiar. However, contrary to H3, there was no interaction between instrument culture and listener group in any of the three context levels.

### Listener-group perception of affective intentions expressed by different instrument categories

As differences in accuracy occur more on an instrument level than as a function of the culture to which the instrument belongs, the data were collapsed across instrument culture, considering only instrument category (blown, bowed, struck) in subsequent analyses and discussion. A three-way interaction between instrument category, listener group, and affective intention addresses the first exploratory point (E1) and would show that particular affective intentions are expressed more effectively in performances by particular categories of instruments and suggest how this might differ between the listener groups. Interaction effects between listener group and affective intentions address a portion of the second exploratory point (E2) and would demonstrate differences between listener groups in their ratings of valence and arousal. The percentages of correct responses for each affective intention in all three contexts are shown in Figure 3(a) to (c) We now present the three factors in turn.

**Figure 3a.**
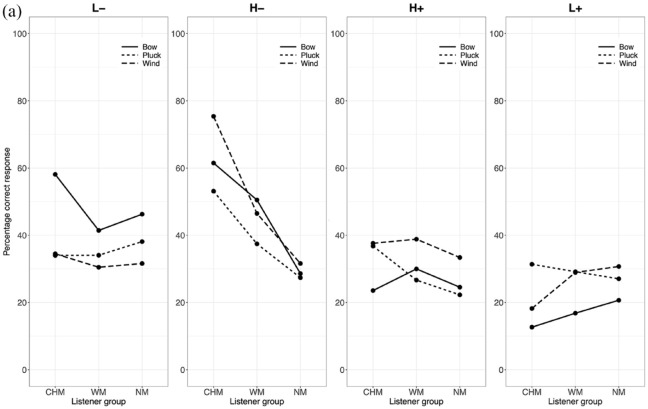
Three-way interaction for the Note context (listener group × affective intention × instrument category).

**Figure 3b.**
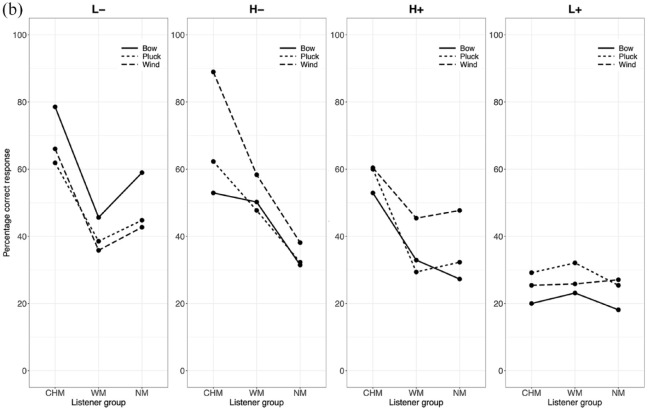
Three-way interaction for the Measure context (listener group × affective intention × instrument category).

**Figure 3c.**
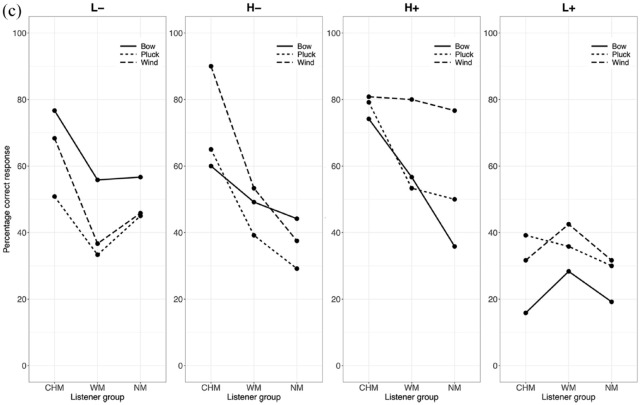
Three-way interaction for the Phrase context (listener group × affective intention × instrument category).

#### Instrument category

Stimuli performed on bowed instruments elicited a higher percentage of accurate responses from all listeners as compared to those performed on plucked or wind instruments for the L− affective intention in all three contexts (Table S2 in Supplementary Materials). The difference in accuracy between stimuli performed on plucked and wind instruments was not statistically significant in any of the three contexts for L− and L+. There was no significant difference in accuracy between stimuli performed on bowed and plucked instruments for H− and H+ in any of the three contexts, and listeners decoded the affective intentions expressed in stimuli performed on wind instruments significantly more accurately than for bowed and plucked instruments in all contexts except for the Note context in H− where bowed and wind instruments were not significantly different. In the H+ affective intention, stimuli performed on wind instruments elicited the largest number of accurate responses from all listeners compared to the other instrument categories across all three contexts. Finally, for the L+ affective intention, stimuli from bowed instruments elicited the lowest percentage of correct responses across all three contexts, but there was no significant difference between those performed on plucked and wind instruments. Performances on bowed instruments were most successful at communicating negatively valenced affective intentions, but not very successful at positively valenced ones, with the exception of H+ in the Phrase context where they were moderately successful. Performances on wind instruments, on the other hand, carried all affective intentions most effectively, except for L+, but especially so for high arousal excerpts in the Measure and Phrase contexts.

#### Listener group

CHM decoded affective intentions more accurately than the other two listener groups for the negatively valenced affective intentions (L− and H−). In the Note context, no significant differences between the listener groups were found for the two positively valenced affective intentions (L+ and H+). For H−, CHM gave more accurate responses than WM, who gave more accurate responses than NM, and for L−, CHM gave more accurate responses than WM. Differences started to appear with increasing context, such that CHM gave more accurate responses than the other two listener groups for L−, H−, and H+ in both Measure and Phrase contexts. In the Measure and Phrase contexts, no differences were observed between WM and NM except for H− in the Measure context where WM were more accurate. Finally, no significant differences in accuracy were observed between the three listener groups for L+ across all three contexts (Supplementary Tables S3a to S3c). Thus, the interaction with context level modulated the main effect of listener group.

#### Affective intention

L+ elicited the lowest percentage of accurate responses across all participants over all three contexts. H+ elicited a significantly lower percentage of accurate responses than H− and L− in the Note and Measure context but a higher percentage than H− and L− in the Phrase context (Supplementary Table S4). A substantial portion of the H+ affective intention was likely communicated more in the note-to-note relationships and less through the quality of the sounds of individual notes. By contrast, H− elicited the highest percentage of accurate responses in the Note context. In the Measure and Phrase contexts, although H− still elicited a higher percentage of accurate responses than H+ and L+, the percentage was not significantly different from that of L−. It is likely that sufficient information about H− can be communicated via individual notes, not requiring more contextual information the way H+ does to accurately communicate its affective intention.

In the Note context, only stimuli performed on wind instruments with the L+ affective intention elicited a significantly higher percentage of accurate responses from NM than CHM, *t*(87) = −2.75, *p* < .05, or WM, *t*(87) = −2.81, *p* < .05. There were no significant differences between the numbers of accurate responses for this affective intention elicited by the three groups in any of the other contexts. In addition, the low number of accurate responses for L+ stimuli as compared to the other affective intentions may be due to L+ sharing acoustic and musical features that made listeners confuse it easily with other affective intentions.

Taken together, these results paint a picture of complex interactions between musical training, affective intention, and the category of instrument used to express the affect. Stimuli performed on bowed instruments appear generally better at communicating low-arousal negatively valenced affective intentions, whereas those on wind instruments communicate high arousal positively valenced affective intentions most effectively. Although CHM generally gave a greater percentage of accurate responses than both WM and NM, and WM gave a greater percentage of accurate responses than NM, there were some instances where this did not hold true. Performance for L+ is difficult to decipher accurately, and musical training does not seem to give listeners an accuracy advantage with this affective intention. Related to this quadrant, peace and tenderness have been found to be confused with sadness or melancholy in music perception studies (e.g., Balkwill & Thompson, 1999; Gabrielsson & Lindström, 2010). Although we specifically used dimensional scales for affect in the present study, the same confusion could still occur as acoustic and musical features might be shared between low-arousal stimuli with positive and negative valence.

### Group differences in valence and arousal ratings

Two-way mixed MANOVA with the between-subjects factor listener group and the within-subject factor instrument were conducted separately for each context (Note, Measure, Phrase) and affective intention (L−, L+, H−, H+) to explore group differences in direct ratings for each affective intention, rather than through the indirect measure of rating accuracy. We determined the effects of these factors on the dependent variables arousal and valence, taking into account the combined effect of the dependent variables on the independent variables. This addresses the second exploratory point (E2). Differences in ratings between the three groups of listeners for each affective intention would suggest that musical culture plays a role in influencing the perception of affective intentions in music independently of how accurately they perceive the performers’ intentions. We observed no consistent patterns in the interactions between instrument culture and category of the performed stimuli, so we took instrument as the independent variable here, with six levels, as it is likely that differences were related to individual instruments rather than the culture or category to which they belong. The average arousal and valence ratings for stimuli performed on each instrument in each affective intention and for the Note, Measure, and Phrase contexts are plotted in Figure 4(a) and (b). Shaded areas indicate the affect intended by the performer. Arousal appeared generally accurate with the data points staying more or less on the correct side of the space (shaded areas). Valence, however, was somewhat more ambiguous; positive valence intentions were sometimes confused with negative valence intentions. With increasing musical context, valence appears to become more differentiated and becomes more accurate for the high arousal intentions. Low-arousal positive-valence (L+), however, continued to be confused with low-arousal negative-valence (L−) even with increased context. Ratings also became more extreme with increased context as listeners probably became more confident of their responses and cues became less ambiguous.

**Figure 4a.**
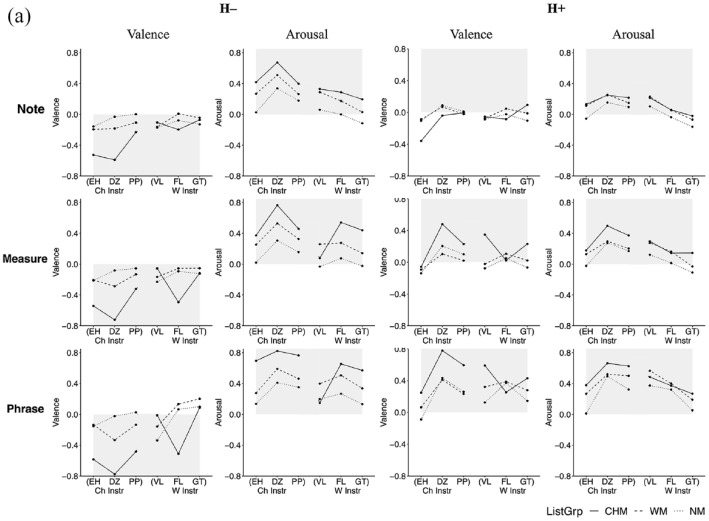
Valence and arousal ratings for high arousal intentions. Note. The shaded area shows intended affect.

**Figure 4b.**
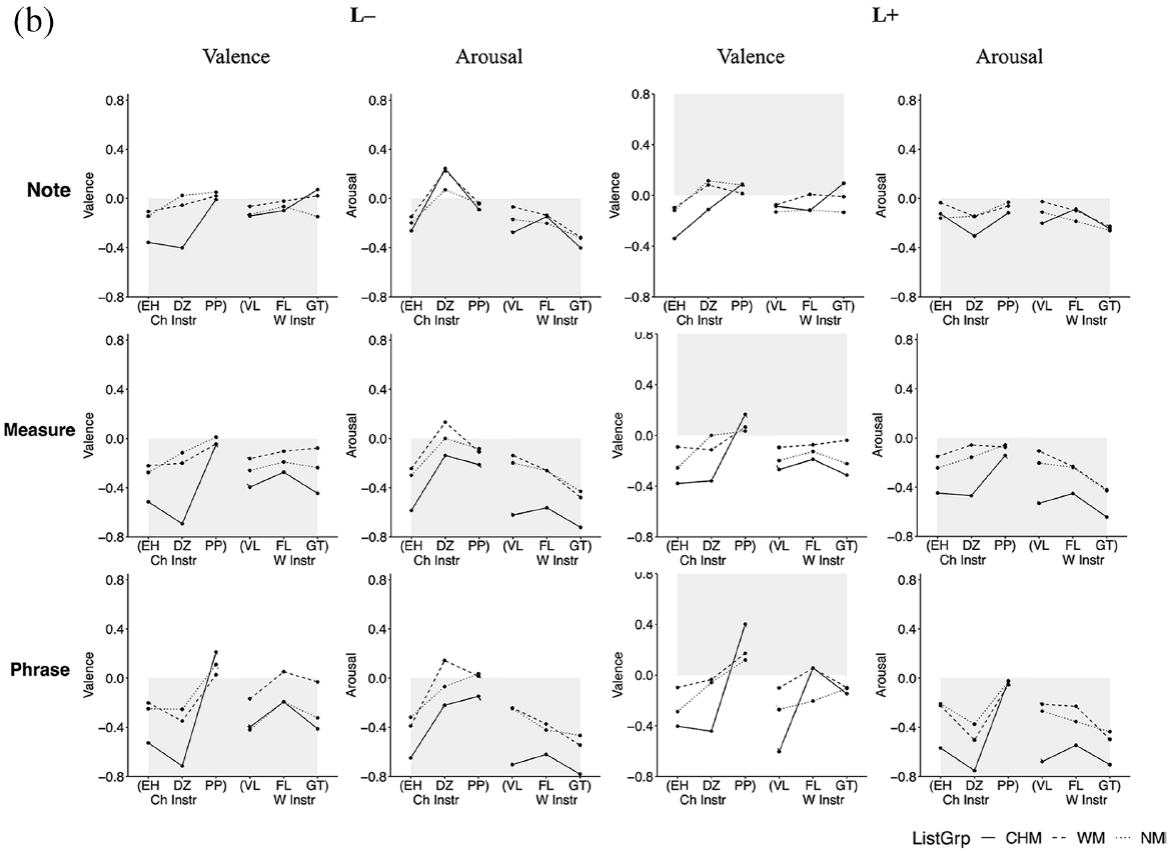
Valence and arousal ratings for low arousal intentions. Note. The shaded area shows intended affect.

The modified ANOVA-type statistic (MATS) was calculated because assumptions of multivariate normality and covariance homogeneity might not be met in the multivariate data obtained in this study. In this statistical test, *p* values were obtained using a parametric bootstrap approach proposed by Konietschke and colleagues (2015) for general MANOVA, and significance was inferred from quantiles of resampling distributions (Friedrich & Pauly, 2018). The main effects of listener group and instrument, as well as the interaction between them, were all statistically significant, indicating that both factors and their interaction are affected by the combined dependent variables. Post hoc ANOVAs with listener group and instrument as independent variables were therefore carried out separately for valence and arousal ratings for each affective intention to examine the results in detail (Supplementary Table S5a to S5c). We corrected all *p* values for multiple comparisons.

#### High arousal negative valence (H−)

The main effects and interactions between them were all significant except for the interaction between listener group and instrument for arousal ratings in the Note context. CHM consistently had the highest arousal ratings followed by WM, and NM had the lowest arousal ratings in this context. With increasing context, this pattern was still observed, except for CHM ratings for H− stimuli performed on the violin, where ratings for arousal decreased and valence became more positive with increasing context. CHM may have been utilizing certain acoustic features prominent in individual notes as cues for high arousal. Although these acoustic features were present in the same amount in the increased contexts, they may have become less important as CHM focused on other musical aspects found in the relationships between notes. For CHM, this focus might have been more salient in the stimuli performed on the violin than on other instruments, but these contextual cues may be in contradiction to the acoustic cues for the H− affective intention. CHM also consistently gave the most negative valence ratings for Chinese instruments and the flute in all three contexts. It appears that this affective intention was communicated very successfully by these instruments to CHM.

#### High arousal positive valence (H+)

There was no significant interaction between the listener group and instrument for arousal ratings in any context for this affective intention. Across all H+ stimuli, CHM generally gave the highest arousal ratings and NM the lowest. For valence ratings in the Note context, CHM gave significantly more negative ratings for stimuli performed on the erhu than did WM and NM. With increasing context, however, CHM valence ratings became significantly different among H+ stimuli performed on all instruments except the flute. It appears that for H+, increasing musical context for stimuli on the erhu, dizi, pipa, violin, and guitar, but not the flute, provided additional information to CHM.

#### Low arousal negative valence (L−)

The main effects and the interactions were all statistically significant for L− stimuli except for the interaction between listener group and instrument for arousal ratings in the Phrase context. CHM tended to give lower or similar valence and arousal ratings compared to the other two listener groups. With increasing context, however, CHM arousal ratings decreased toward lower arousal and more negative valence, whereas WM and NM arousal ratings remained high. Increased musical context appeared to provide the relevant information for this affective intention to a greater extent for CHM than the other two groups.

#### Low arousal positive valence (L+)

There were significant main effects and interactions between them for all three contexts in L+ . Listeners found it difficult to recognize the positive valence intended to be communicated by L+ stimuli performed on all instruments except the pipa. The average valence ratings of all three groups of listeners were in the negative range and did not appear to increase even with increasing musical context. Low arousal was communicated successfully on the L+ stimuli, but positive valence was conveyed in L+ only in stimuli performed on the pipa.

### Partial least-squares regression of acoustic features with affect ratings

Using the revised Timbre Toolbox (Kazazis et al., 2021) implemented in the MATLAB environment, temporal, spectral, and spectrotemporal features of individual notes were analyzed. Based on hierarchical clustering analyses done by Peeters and colleagues (2011) with the original version of the toolbox, we selected 13 acoustic features that represent the different clusters. These acoustic features included medians (central tendencies) and interquartile ranges (IQRs, variability over time) of spectral centroid, spectral flatness, and RMS envelope, as well as the median for noisiness, harmonic spectral deviation, spectral variation, temporal centroid, and frequency and amplitude of energy modulation. Log attack time was also included. These calculations were performed on individual notes. For measures and phrases, the average value of the feature across all notes included in the measure or phrase was used.

We explored the relationship between acoustic features and valence and arousal ratings using a PLSR analysis. The PLSR examined the relationship between acoustic features and different listener groups’ ratings of valence and arousal. Each listener group was examined separately to see if there were differences in the acoustic features that might predict their ratings of perceived affective intentions. A greater number of overlapping acoustic features loading onto each principal component that are shared between CHM and WM as compared to NM would support the fourth hypothesis (H4) that CHM and WM use similar acoustic cues for decoding affective intentions, whereas those used by NM are different. An examination of the acoustic features that load onto each principal component would also provide information about the timbral properties that inform listeners’ perceptions of different affective intentions, allowing us to address our third exploratory point (E3).

A 10-fold cross-validation model was applied to the PLSR model with training on nine subsets and testing on the remaining one to estimate prediction error. PLSR statistics were done using the *prcomp* package in R 3.6.2 (R Core Team, 2022). The number of latent variables was selected by taking the point where the drop in the root mean squared error of the prediction score after cross-validation was no longer significant. A VARIMAX rotation was performed on the factors to simplify the structure and improve explanatory power. This yielded different numbers of latent variables for the different listener groups. The percentage of variance explained by each component is shown on the loading plots. Figure 5(a) to (c) visualizes the loadings of the components for valence and arousal in CHM, WM, and NM listeners, respectively. Table 1 lists the factors contributing to each principal component with their loadings. Only factors with loadings of 0.2 or greater are listed.

**Figure 5a.**
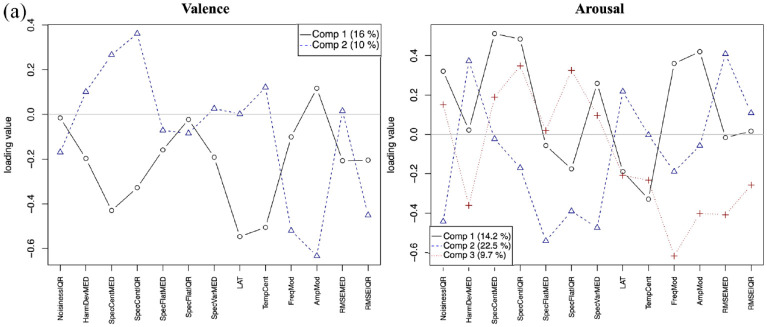
Loadings of audio descriptors for valence and arousal for CHM.

**Figure 5b.**
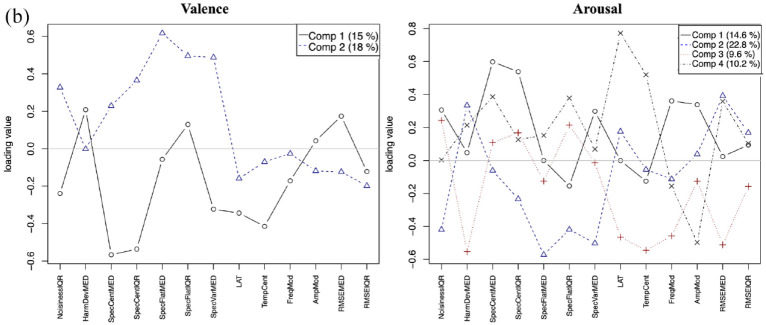
Loadings of audio descriptors for valence and arousal for WM.

**Figure 5c.**
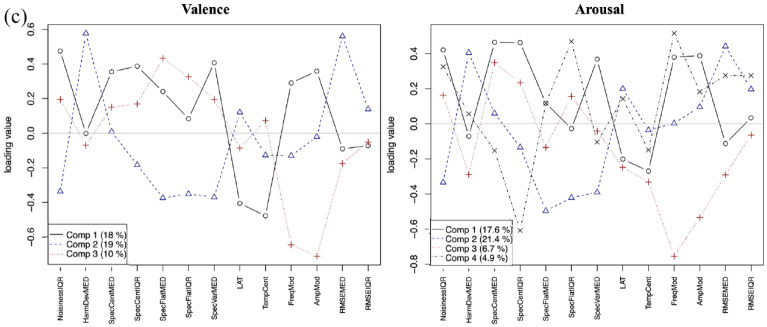
Loadings of audio descriptors for valence and arousal for NM.

**Table 1.** Loadings for valence and arousal on each principal component for Chinese musicians (CHM), Western musicians (WM), and nonmusicians (NM).

		Valence		Arousal	
CHM	PC1	Spectral centroid (med) Spectral centroid (IQR) Log attack time Temporal centroid RMS energy (med) RMS energy (IQR)	−0.430 −0.328 −0.547 −0.505 −0.207 −0.205	Noisiness (IQR) Spectral centroid (med) Spectral centroid (IQR) Spectral variation (med) Temporal centroid Modulation frequency Modulation amplitude	0.321 0.512 0.484 0.259 −0.329 0.360 0.420
	PC2	Spectral centroid (med) Spectral centroid (IQR) Modulation frequency Modulation amplitude RMS energy (IQR)	0.267 0.362 −0.520 −0.633 −0.451	Noisiness (IQR) Harmonic deviation (med) Spectral flatness (med) Spectral flatness (IQR) Spectral variation (med) Log attack time RMS energy (med)	−0.442 0.373 −0.541 −0.390 −0.475 0.218 0.408
	PC3			Harmonic deviation (med) Spectral centroid (IQR) Spectral flatness (IQR) Log attack time Temporal centroid Modulation frequency Modulation amplitude RMS energy (med) RMS energy (IQR)	−0.361 0.348 0.326 −0.208 −0.233 −0.618 −0.402 −0.409 –0.258
WM	PC1	Noisiness (IQR) Harmonic deviation (med) Spectral centroid (med) Spectral centroid (IQR) Spectral variation (med) Log attack time Temporal centroid	−0.239 0.209 −0.566 −0.536 −0.323 −0.343 −0.415	Noisiness (IQR) Spectral centroid (med) Spectral centroid (IQR) Spectral variation (med) Modulation frequency Modulation amplitude	0.306 0.598 0.538 0.298 0.361 0.338
	PC2	Noisiness (IQR) Spectral centroid (med) Spectral centroid (IQR) Spectral flatness (med) Spectral flatness (IQR) Spectral variation (med)	0.328 0.229 0.365 0.618 0.496 0.488	Noisiness (IQR) Harmonic deviation (med) Spectral centroid (IQR) Spectral flatness (med) Spectral flatness (IQR) Spectral variation (med) RMS energy (med)	−0.420 0.334 −0.234 −0.572 −0.419 −0.502 0.392
	PC3			Noisiness (IQR) Harmonic deviation (med) Spectral flatness (IQR) Log attack time Temporal centroid Modulation frequency RMS energy (med)	0.243 −0.553 0.214 −0.464 −0.545 −0.458 −0.511
	PC4			Harmonic deviation (med) Spectral centroid (med) Spectral flatness (IQR) Log attack time Temporal centroid Modulation amplitude RMS energy (med)	0.214 0.386 0.378 0.772 0.520 −0.497 0.359
NM	PC1	Noisiness (IQR) Spectral centroid (med) Spectral centroid (IQR) Spectral flatness (med) Spectral variation (med) Log attack time Temporal centroid Modulation frequency Modulation amplitude	0.475 0.355 0.387 0.241 0.407 −0.405 −0.477 0.291 0.359	Noisiness (IQR) Spectral centroid (med) Spectral centroid (IQR) Spectral variation (med) Log attack time Temporal centroid Modulation frequency Modulation amplitude	0.421 0.464 0.463 0.368 −0.201 −0.270 0.380 0.388
	PC2	Noisiness (IQR) Harmonic deviation (med) Spectral flatness (med) Spectral flatness (IQR) Spectral variation (med) RMS energy (med)	−0.336 0.577 −0.374 −0.351 −0.370 0.560	Noisiness (IQR) Harmonic deviation (med) Spectral flatness (med) Spectral flatness (IQR) Spectral variation (med) Log attack time RMS energy (med)	−0.334 0.405 −0.496 −0.421 −0.390 0.200 0.442
	PC3	Spectral flatness (med) Spectral flatness (IQR) Modulation frequency Modulation amplitude	0.433 0.325 −0.645 −0.709	Harmonic deviation (med) Spectral centroid (med) Spectral centroid (IQR) Log attack time Temporal centroid Modulation frequency Modulation amplitude RMS energy (med)	−0.289 0.349 0.234 −0.248 −0.332 −0.754 −0.534 −0.291
	PC4			Noisiness (IQR) Spectral centroid (IQR) Spectral flatness (IQR) Modulation frequency RMS energy (med) RMS energy (IQR)	0.325 −0.608 0.469 0.515 0.276 0.275

The acoustic features characterize different aspects of a sound. The mapping from the acoustic space to a semantic description of the qualities of the sounds could be somewhat ambiguous, but some authors have found terms that appear to be relatively consistent (Saitis & Weinzierl, 2019; Wallmark & Kendall, 2018; Zacharakis et al., 2012). In the exploration of acoustic features informing listeners’ perception of affective intentions that follow, rather than simply listing all the relevant acoustic features for each condition from Table 1, we will describe the qualities of the sounds reflected by the ways in which the combinations of acoustic features map onto adjectives commonly found in the timbre semantics literature.

#### Valence

##### Chinese musician listeners

Two components explained the loadings for valence (16% for PC1 with 6 acoustic features, 10% for PC2 with 5 features, 26% total variance explained; Figure 5[a], left panel). It appeared that CHM listeners perceive darker, softer, and less sustained sounds as more positively valenced (PC1). However, if the sounds are bright but combined with less vibrato and little change in dynamics (PC2), CHM also perceived them as communicating positive valence.

##### Western musician listeners

Two components also explained the loadings for valence (15% for PC1 with 7 features, 18% for PC2 with 6 features, 33% total; Figure 5[b], left panel). Similar to CHM, a darker and less sustained sound with less spectral variation communicated positive valence (PC1). When the sound is bright and combined with large spectral variation, and is high in noise content (PC2), WM perceived it as positively valenced. WM shared four of the seven acoustic features loading onto the first principal component and two of the six features loading on the second component with CHM. Thus, a different combination of features cued positive valence for WM. This analysis accounts for the shared similarities in some of the valence responses with CHM but divergences in others.

##### Nonmusician listeners

Three components explained the loadings for valence (18% for PC1 with nine features, 19% for PC2 with six features, 10% for PC3 with four features, and 47% total; Figure 5[c], left panel). NM listeners shared five of the nine features on their first component with WM’s second component and only two features with CHM’s second component. Their second component only shared three of its six features with WM’s first component. They were distinguished by a third component with four features. Although NM perceived variability in noisiness, brightness, and large spectral variation as communicating positive valence, they also combined this set of features with a less sustained sound and a large amount of vibrato (PC1). This particular combination differed from CHM who used the combination of a large amount of vibrato with brightness to imply negative valence. It also differed from WM whose combination of less sustained sounds with lower brightness and spectral variation implied positive valence. Altogether, these results point toward more shared commonalities between NM and WM than with CHM, which were reflected both in the accuracy and pattern of responses of the three listener groups, but hypothesis H4 was not supported.

#### Arousal

Globally, arousal ratings had more principal components involving a wider variety of acoustic features for all three listener groups than did valence ratings. This result suggests a more complex acoustic basis for valence than arousal perception and also perhaps a closer link between arousal and sound properties than more culturally determined factors.

##### Chinese musician listeners

Three components explained the loadings for arousal (14.2% for PC1 with seven features, 22.5% for PC2 with seven features, 9.7% for PC3 with nine features, and 46.4% total) in CHM (Figure 5[a], right panel). A brighter sound with more spectral variation, noisiness, and vibrato pointed toward higher arousal for CHM (PC1). A louder sound that is less noisy implies higher arousal (PC2). A brighter sound with more vibrato that is also less sustained might be perceived as higher in arousal (PC3).

##### Western musician listeners

Four components explained the variance for arousal (14.6% for PC1 with six features, 22.8% for PC2 with seven features, 9.6% for PC3 with seven features, 10.2% for PC4 with seven features, and 57.2% total) in WM (Figure 5[b], right panel). Brighter and noisier sounds with more vibrato and spectral variation were perceived as higher in arousal (PC1), whereas sounds that are loud but have less noise content were also perceived as high in arousal (PC2). Shorter, less sustained sounds with little vibrato communicated high arousal as well (PC3), but the combination of loudness, brightness, and more sustained sounds also communicated high arousal (PC4). WM shared all six features on PC1, six of the seven features on PC2, and six of the seven features on PC3 with CHM but had an additional principal component.

##### Nonmusician listeners

Four components explained the loadings for arousal (17.6% for PC1 with eight features, 21.4% for PC2 with seven features, 6.7% for PC3 with eight features, 4.9% for PC4 with six features, and 50.6% total) in the NM listeners (Figure 5[c], right panel). Similarly to the other groups of listeners, a brighter sound with high noise content, substantial vibrato, and large spectral variation generally indicated a higher arousal (PC1). A combination of loudness, less noise content, and less spectral variation also communicated high arousal similarly for all three groups of listeners (PC2). Brighter, shorter, less sustained sounds with little vibrato also implied high arousal for all three groups (PC3). NM shared seven of the eight features with CHM and six with WM on PC1. On PC2, they shared all seven features with CHM and five features with WM. On PC3, NM shared seven of the eight features with CHM and five with WM. PC4 distinguished this group from the other two.

##### Summary

Results from the PLSR demonstrate clearly that individual acoustic features do not make independent contributions to the perception of valence or arousal. Many acoustic features loaded positively in one principal component but negatively in another. This is likely due to interactions between the different acoustic features, with affective intentions being elicited by combinations of acoustic features rather than single features. The acoustic features contributing to valence were much more influenced by musical training and musical tradition than those that contributed to arousal. In contrast with hypothesis H4, NM and WM shared a small number of similar acoustic features they used to decode valence and both groups shared fewer features with CHM. Arousal, by contrast, appeared to be elicited by more similar combinations of acoustic features for all three groups.

A larger percentage of the variance could be explained by acoustic features for arousal than for valence, with 26%, 33%, and 47% for valence and 46.4%, 57.2%, and 60.6% for arousal in CHM, WM, and NM, respectively. This result suggests that in addition to surface acoustic features, other factors could be at play in communicating perceived valence to listeners, more so than for perceived arousal. Similarly, a larger percentage of the variance for both dimensions was explained by acoustic features for NM, followed by WM, and the smallest percentage for CHM. It appears there were aspects of the musical sound that might not be captured by these acoustic features but which cued listeners as to its perceived affective intention. Furthermore, the ways in which these aspects were utilized were influenced by musical training.

## Discussion

In this study, we examined the influences of training in different musical cultures on listeners’ perception of affective intentions in music and explored the ways in which listeners with different musical trainings utilize acoustic cues in their perception of affective intentions.

### Differences in listeners’ responses

Average ratings of perceived affective intentions for each group of listeners showed a general trend of becoming more differentiated as context increased from Notes to Measures to Phrases. Arousal ratings tended to be more accurate than valence ratings, although the pattern was different for each instrument and each group of listeners. These results are consistent with Egermann and colleagues’ (2015) finding that arousal responses seem to be based more on culturally invariant cues or mechanisms, whereas valence responses are more culture-dependent.

Confirming our hypothesis H1, participants showed a significant increase in the percentage of accurate responses as context increased, with a large main effect for context, and their ratings also became more differentiated. The three listener groups were also significantly different in terms of percentage of response accuracy (CHM > WM > NM), and this main effect showed a large effect size partially in line with hypothesis H2 that musicians perform better than nonmusicians, although CHM did outperform WM. The reason for this difference could be because of contrasting emphasis on the use of timbre in the musical training of the Chinese as compared to the Western musical tradition, which allowed Chinese musicians (CHM) to be sensitive to minute changes in the way timbre is manipulated in the expression of affective intentions. Thompson and Balkwill’s (2010) cue-redundancy model states that “listeners who are familiar with a musical style should find it relatively easy to decode emotional meaning in that music, because they can draw from both culture-specific and psychophysical cues” (p. 766). This would mean that Western musicians (WM) decode stimuli interpreted by Western instruments with greater accuracy and CHM decode stimuli interpreted by Chinese instruments with greater accuracy. However, this was not observed, refuting hypothesis H3: there did not seem to be a cultural advantage for WM, as CHM consistently had a larger percentage of accurate responses than WM and NM regardless of the instrument tradition of the stimuli.

Both Chinese orchestral music and Western classical music are ubiquitous musical traditions in Singapore, and CHM, WM, and NM had a similar amount of casual exposure to these musics in their everyday life. The only difference then would appear to be formal training and possibly the conscious choice of listeners to listen to one particular type of music over another. The majority of CHM also did not have any formal training in Western music so the difference in accuracy is unlikely to be due to CHM having a broader range of casual or formal musical experiences as compared to WM. In addition to that, these results suggest that CHM had access to more cues for affective intentions than WM or NM because of their greater sensitivity to timbral cues. In the Note context, musical relations were not as available for the listener, and responses were based almost entirely on the perceived timbre of the individual note. CHM might have been more sensitive to the nuances of timbre variation from the emphasis on it in their musical training, whereas knowledge of musical relations habituated by musical training provided additional cues for both the musician groups with increasing musical context. Although stimuli performed on Chinese instruments were marginally better than Western instruments in eliciting accurate responses about their expressed affective intention in the Note context, there were no differences between the instrument cultures in the other two contexts. Similarly, performers on Chinese instruments in this study might have placed greater emphasis on the timbral features within each note when expressing affective intentions.

Certain instrumental timbres might be better at expressing one affect than others, possibly due not only to the characteristics of the instrument, but also to its connotations and use in particular musical tropes. For example, Behrens and Green (1993) found that for Western listeners, performances on the violin were able to express sadness more accurately than other instruments. This appeared to be the case in this study as well, with stimuli performed on the violin and erhu eliciting the highest percentage of correct responses in the low-arousal negative-valence intention. Hailstone and colleagues (2009) also note that “the perception of emotion conveyed by a melody is affected by the identity or timbre of the musical instrument on which it is played” (p. 2152). Composers appear to understand this implicitly, selecting particular instruments over others more frequently to express certain emotions (e.g., Huron et al., 2014; Schutz et al., 2008). In addition to affordances and constraints of instruments for the expression of affective intentions, performer variability could play a role as well. It was not possible in the context of this study to explore variability between performers, or to control for individual variation in performances on different instruments. To reduce influence from this as much as is possible, all the excerpts were interpreted by musicians who perform regularly on a professional basis. This may have also allowed, however, for idiosyncratic interpretations of the affective intentions of a particular instrument. With regards to exploration point E1, the results suggested that instead of stimuli performed by categories of instruments (blown, bowed, plucked) that might be more effective at expressing particular affective intentions, differences appeared more on the individual instrument level. Stimuli expressing H− performed on the erhu, dizi, pipa, violin, and guitar, for instance, elicited the highest arousal and most negative valence ratings for CHM as compared to the other two listener groups, whereas stimuli expressing H+ performed on the flute elicited the highest arousal and most positive valence ratings for WM as compared to the other two listener groups.

With regards to exploration point E2, we found that accuracy varied across the different affective intentions. The listener group × affective intention interaction showed a medium size of effect. The MANOVA also showed that CHM were generally more accurate and had more extreme ratings than the other two listener groups in high arousal negative-valence intentions. Susino and Schubert (2017) proposed a stereotype theory of emotion to explain how perceptions of emotions might be filtered through a listener’s stereotype of the encoding culture. East Asians are stereotyped as significantly less emotionally expressive than European Americans (Adam & Shirako, 2013). WM might believe that Chinese music is less likely to express anger. This might partially explain why WM perceived less of the stimuli performed by Chinese instruments as high arousal negative valence. In addition, it is also likely that there is a highly nuanced set of timbre characteristics for this affective intention that is used in the Chinese music tradition, which CHM understood and were able to use effectively in their listening responses. The low-arousal positive-valence intention appeared to be the least accurate in all the three listener groups, and CHM gave the lowest percentage of accurate responses, a response pattern that was in contrast with all the other affective intentions in which CHM were usually the most accurate. It could be that low-arousal positive-valence intentions shared timbral and musical characteristics with other affective intentions, making it difficult to distinguish them from the others, not only in the single-note context but also in added musical context. This is consistent with other studies that found tenderness and peace to be correlated with sadness (e.g., Balkwill & Thompson, 1999; Gabrielsson & Lindström, 2010).

### Effects of timbre on perception of valence and arousal

Results of the PLSR analysis of acoustic features with valence and arousal ratings helped to demonstrate the contributions of these features to listeners’ perceptions of valence and arousal for exploration point E3. Rather than features contributing to an affective intention independently, it appeared that the perception of affective intentions was influenced by a combination of various acoustic features. Features also do not necessarily map onto affect perception in the same direction. A particular acoustic feature might map in different directions for particular affective intentions depending on other features it interacts with.

Consistent with the findings of Egermann et al. (2015), it also appears that listeners’ use of these features for valence perception was very much influenced by musical training and enculturation, and there was only a very small overlap in the acoustic cues used by the three groups. In contrast, the three groups of listeners shared more of the same combinations of acoustic features that they used to decode arousal levels. An understanding of the acoustic features that contribute to the communication of arousal may be more culturally invariant, whereas those that communicate valence could be more culturally learned. Hypothesis H4 is therefore not supported by the data.

Additionally, it appears that other factors besides surface acoustic features play a greater role in explaining the variance for perceived valence than arousal. Similarly, surface acoustic features explained the largest amount of the variance in the PLSR for NM and the least for CHM. Even though all the participants were presented with the same acoustic stimuli, different listener groups used different amounts and types of cues for decoding affective intentions. When these features aligned in production and comprehension with the intended affect, listeners were better at recognizing the intended emotion. However, even NM was not totally unsuccessful in decoding affective intentions. There is a redundancy of cues in musical communication, and a certain degree of emotional recognition is still possible even when several cues are misaligned.

It is worth mentioning some limitations of the study. Only one excerpt was used in the experiment, and only one performer for each instrument to keep the duration of the experimental sessions manageable and avoid participant fatigue. In future, more performers could be recruited to play a range of excerpts on each instrument. This would provide better control for the affective tendencies that may be intrinsic to any given melody. Different performers on each instrument would also provide better control for individual variability in performance.

The instruments the musician participants play have not been systematically compared to their response accuracy for the stimuli performed by different instruments although there is a general representation of the various instrument families with regards to the instruments they play. This point would be worth exploring in future studies to see if expertise in certain instruments might improve response accuracy in stimuli performed by those instruments.

No continuous response was elicited from listeners with respect to changes in affective intentions over the course of the excerpts in this study. Although the comparisons across responses for Notes, Measures, and Phrases provide an indication of musical context providing increasing cues for understanding, researchers could attempt to look at continuous responses in future studies to better understand the function of timbre over the course of the music. Participants with formal training in other musical cultures could also be recruited to explore the perception of affective intentions and timbre by listeners from different musical traditions.

## Conclusion

The function of timbre in communicating affective intention in music is highly complex. There are many interactions between the acoustic features that make up the quality of a sound. Listeners make use of the relationship between these acoustic features and other musical parameters such as pitch relations, implied harmonies, and rhythmic and metrical characteristics, with increasing musical context. Listeners from different musical traditions perceive affective intentions with differing degrees of accuracy, suggesting an important role for musical tradition as well as musical training in listener responses. In the context of this study, Chinese musicians were consistently more accurate than both Western musicians and nonmusicians, and this may be related to the differences between Chinese and Western musical traditions with regards to the use of timbre in expression. Anecdotal experience reveals that in the Chinese musical tradition there is often very explicit discussions about affective intentions and ways of realizing the music in performance. Because the former relies more on certain means of timbral expression to convey particular affective intentions, Chinese musicians are likely to be more sensitive to nuances when listening to music. Although this gives Chinese musicians an advantage because they have more cues to help them decode affective intentions accurately, the abundance and indeed redundancy of psychophysical cues in music enables even nonmusicians to recognize emotions to some degree at least.

The acoustic features that elicit arousal responses appear to be more similar across the three listener groups than those that elicit valence responses. This result strongly suggests that conventions regarding the function of timbre in valence perception are learned through explicit musical training and culture-specific experience, whereas those for arousal might be more culturally invariant. Also, particular intentions are not conveyed by individual acoustic features but, rather, listeners perceive the entirety of the timbre of a sound through various combinations of acoustic features.

There might have been a slight asymmetry in participant expertise given that four of the 30 Chinese musician participants had had formal instruction in Western music, but none of the Western musician listeners had had any formal instruction in Chinese music. There were so few CHM listeners with formal training on a Western instrument, however, and the duration of their training was so short that this asymmetry is unlikely to have influenced the results of the study. In future studies it would be worth exploring potential differences between Chinese musician listeners with and without formal training in Western music. It should also be acknowledged that we recruited participants on the basis of how long they had undergone formal training in a particular musical tradition. We did not therefore consider potential differences between individuals in terms of the extent to which they may have absorbed musical conventions implicitly through exposure. In future studies, it would be worth administering validated measures, adapted as appropriate, of participants’ musical exposure and knowledge.

The findings of this study demonstrate the roles played by cultural and formal musical learning in timbre perception. Until now, most studies have recruited Western participants and used Western musical instruments and proto-musical materials or music from the Western classical repertoire. Our use of Chinese instruments provides a different perspective on music perception. In addition, we recruited participants from the same geographical location with different kinds of formal musical training. In this way, we reduced the likelihood that differences between groups would be attributable to socio-cultural or linguistic factors.

## Supplemental Material

sj-docx-1-msx-10.1177_10298649241237775 – Supplemental material for The function of timbre in the perception of affective intentions: Effect of enculturation in different musical traditions Supplemental material, sj-docx-1-msx-10.1177_10298649241237775 for The function of timbre in the perception of affective intentions: Effect of enculturation in different musical traditions by Lena Heng and Stephen McAdams in Musicae Scientiae
