# Supplementary material for: The function of timbre in the perception of affective intentions: Effect of enculturation in different musical traditions
Source: Music Sci. 2024 Mar 18;28(4):675–702. doi: 10.1177/10298649241237775 (PMC11560474; doi:10.1177/10298649241237775)
Supplement: sj-docx-1-msx-10.1177_10298649241237775 – Supplemental material for The function of timbre in the perception of affective intentions: Effect of enculturation in different musical traditions [file sj-docx-1-msx-10.1177_10298649241237775.docx]

Table S1a: Four-way ANOVA on rank transformed data for Note accuracy.

|  | **Df** | **Df.res** | **F** | ***p*** | **partial η^2^** | **signif.** |
| --- | --- | --- | --- | --- | --- | --- |
| Listener Group (ListGrp) | 2 | 87 | 62.24 | < .001 | .59 | *** |
| Affective Intention (AffInt) | 3 | 2001 | 158.31 | < .001 | .19 | *** |
| Instrument Culture (InstrCult) | 1 | 2001 | 12.80 | < .001 | .006 | *** |
| Instrument Category (InstrCat) | 2 | 2001 | 6.01 | .0025 | .006 | ** |
| ListGrp:AffInt | 6 | 2001 | 44.96 | < .001 | .12 | *** |
| ListGrp:InstrCult | 2 | 2001 | 1.05 | .35 | .001 |  |
| AffInt:InstrCult | 3 | 2001 | 45.31 | < .001 | .06 | *** |
| ListGrp:InstrCat | 4 | 2001 | 0.60 | .66 | .001 |  |
| AffInt:InstrCat | 6 | 2001 | 33.59 | < .001 | .09 | *** |
| InstrCult:InstrCat | 2 | 2001 | 7.14 | < .001 | .007 | *** |
| ListGrp:AffInt:InstrCult | 6 | 2001 | 8.58 | < .001 | .02 | *** |
| ListGrp:AffInt:InstrCat | 12 | 2001 | 7.28 | < .001 | .04 | *** |
| ListGrp:InstrCult:InstrCat | 4 | 2001 | 0.38 | .82 | .0008 |  |
| AffInt:InstrCult:InstrCat | 6 | 2001 | 18.28 | < .001 | .05 | *** |
| ListGrp:AffInt:InstrCult:InstrCat | 12 | 2001 | 1.59 | .089 | .009 |  |

Signif. codes: *** .001 **.01 *.05

|  | **Df** | **Df.res** | **F** | ***p*** | **partial η^2^** | **signif.** |
| --- | --- | --- | --- | --- | --- | --- |
| Listener Group (ListGrp) | 2 | 87 | 82.64 | < .001 | .66 | *** |
| Affective Intention (AffInt) | 3 | 2001 | 185.23 | < .001 | .22 | *** |
| Instrument Culture (InstrCult) | 1 | 2001 | 3.54 | .06 | .002 |  |
| Instrument Category (InstrCat) | 2 | 2001 | 18.02 | < .001 | .02 | *** |
| ListGrp:AffInt | 6 | 2001 | 33.05 | < .001 | .09 | *** |
| ListGrp:InstrCult | 2 | 2001 | 0.09 | .91 | .00009 |  |
| AffInt:InstrCult | 3 | 2001 | 38.72 | < .001 | .05 | *** |
| ListGrp:InstrCat | 4 | 2001 | 1.76 | .13 | .004 |  |
| AffInt:InstrCat | 6 | 2001 | 20.44 | < .001 | .06 | *** |
| InstrCult:InstrCat | 2 | 2001 | 7.77 | < .001 | .008 | *** |
| ListGrp:AffInt:InstrCult | 6 | 2001 | 1.64 | .13 | .005 |  |
| ListGrp:AffInt:InstrCat | 12 | 2001 | 4.91 | < .001 | .03 | *** |
| ListGrp:InstrCult:InstrCat | 4 | 2001 | 0.48 | .75 | .001 |  |
| AffInt:InstrCult:InstrCat | 6 | 2001 | 30.82 | < .001 | .08 | *** |
| ListGrp:AffInt:InstrCult:InstrCat | 12 | 2001 | 8.15 | < .001 | .05 | *** |

Table S1b: Four-way ANOVA on rank transformed data for Measure accuracy.

Signif. codes: *** .001 **.01 *.05

Table S1c: 4-way ANOVA on rank transformed data for Phrase accuracy.

|  | **Df** | **Df.res** | **F** | ***p*** | **partial η^2^** | **signif.** |
| --- | --- | --- | --- | --- | --- | --- |
| Listener Group (ListGrp) | 2 | 87 | 50.45 | < .001 | .54 | *** |
| Affective Intention (AffInt) | 3 | 2001 | 92.85 | < .001 | .12 | *** |
| Instrument Culture (InstrCult) | 1 | 2001 | 0.22 | .64 | .0001 |  |
| Instrument Category (InstrCat) | 2 | 2001 | 21.44 | < .001 | .02 | *** |
| ListGrp:AffInt | 6 | 2001 | 11.20 | < .001 | .03 | *** |
| ListGrp:InstrCult | 2 | 2001 | 3.65 | .03 | .004 | * |
| AffInt:InstrCult | 3 | 2001 | 19.50 | < .001 | .03 | *** |
| ListGrp:InstrCat | 4 | 2001 | 1.02 | .40 | .002 |  |
| AffInt:InstrCat | 6 | 2001 | 14.39 | < .001 | .04 | *** |
| InstrCult:InstrCat | 2 | 2001 | 2.99 | .05 | .003 |  |
| ListGrp:AffInt:InstrCult | 6 | 2001 | 3.42 | .002 | .01 | ** |
| ListGrp:AffInt:InstrCat | 12 | 2001 | 3.86 | < .001 | .02 | *** |
| ListGrp:InstrCult:InstrCat | 4 | 2001 | 4.93 | < .001 | .01 | *** |
| AffInt:InstrCult:InstrCat | 6 | 2001 | 18.66 | < .001 | .05 | *** |
| ListGrp:AffInt:InstrCult:InstrCat | 12 | 2001 | 3.06 | < .001 | .02 | *** |

Signif. codes: *** .001 **.01 *.05

Table S2. Post hoc comparisons for instrument category across all listener groups and contexts

| **Affective intention: low arousal, negative valence (L-)** | ***df*** | ***t* ratio** | ***p* value** |
| --- | --- | --- | --- |
| Bow,Note - Pluck,Note | 1506 | 5.27 | < .001 |
| Bow,Note - Wind,Note | 1506 | 6.65 | < .001 |
| Pluck,Note - Wind,Note | 1506 | 1.37 | 1.0000 |
| Bow,Measure - Pluck,Measure | 1506 | 5.02 | < .001 |
| Bow,Measure - Wind,Measure | 1506 | 4.70 | < .001 |
| Pluck,Measure - Wind,Measure | 1506 | –0.32 | 1.0000 |
| Bow,Phrase - Pluck,Phrase | 1506 | 6.99 | < .001 |
| Bow,Phrase - Wind,Phrase | 1506 | 4.63 | < .001 |
| Pluck,Phrase - Wind,Phrase | 1506 | –2.36 | .1831 |
| **Affective intention: high arousal, negative valence (H-)** |  |  |  |
| Bow,Note - Pluck,Note | 1506 | 2.70 | .1327 |
| Bow,Note - Wind,Note | 1506 | –1.39 | 1.0000 |
| Pluck,Note - Wind,Note | 1506 | –4.09 | .001 |
| Bow,Measure - Pluck,Measure | 1506 | –1.14 | 1.0000 |
| Bow,Measure - Wind,Measure | 1506 | –6.03 | < .001 |
| Pluck,Measure - Wind,Measure | 1506 | –4.89 | < .001 |
| Bow,Phrase - Pluck,Phrase | 1506 | 2.42 | .2689 |
| Bow,Phrase - Wind,Phrase | 1506 | –3.28 | .0232 |
| Pluck,Phrase - Wind,Phrase | 1506 | –5.70 | < .001 |

Table S2 (cont.). Post hoc comparisons for instrument category across all listener groups and contexts

| **Affective intention: high arousal, positive valence (H+)** | ***df*** | ***t* ratio** | ***p* value** |
| --- | --- | --- | --- |
| Bow,Note - Pluck,Note | 1506 | –1.26 | .63 |
| Bow,Note - Wind,Note | 1506 | –4.79 | < .001 |
| Pluck,Note - Wind,Note | 1506 | –3.54 | .004 |
| Bow,Measure - Pluck,Measure | 1506 | –1.45 | .58 |
| Bow,Measure - Wind,Measure | 1506 | –4.96 | < .001 |
| Pluck,Measure - Wind,Measure | 1506 | –3.50 | .004 |
| Bow,Phrase - Pluck,Phrase | 1506 | –2.56 | .06 |
| Bow,Phrase - Wind,Phrase | 1506 | –8.59 | < .001 |
| Pluck,Phrase - Wind,Phrase | 1506 | –6.03 | < .001 |
| **Affective intention: low arousal, positive valence (L+)** |  |  |  |
| Bow,Note - Pluck,Note | 1506 | –5.63 | < .001 |
| Bow,Note - Wind,Note | 1506 | –4.05 | .001 |
| Pluck,Note - Wind,Note | 1506 | 1.57 | 1.0000 |
| Bow,Measure - Pluck,Measure | 1506 | –3.80 | .004 |
| Bow,Measure - Wind,Measure | 1506 | –2.90 | .07 |
| Pluck,Measure - Wind,Measure | 1506 | 0.91 | 1.0000 |
| Bow,Phrase - Pluck,Phrase | 1506 | –6.05 | < .001 |
| Bow,Phrase - Wind,Phrase | 1506 | –5.63 | < .001 |
| Pluck,Phrase - Wind,Phrase | 1506 | 0.42 | 1.0000 |

Table S3a. Post hoc comparisons for listener groups across all instruments for the Note context

| **Affective intention: low arousal, negative valence (L-)** | ***df*** | ***t* ratio** | ***p* value** |
| --- | --- | --- | --- |
| CHM - WM | 87 | 2.69 | .03 |
| CHM - NM | 87 | 1.84 | .14 |
| WM - NM | 87 | –0.85 | .40 |
| **Affective intention: high arousal, negative valence (H-)** |  |  |  |
| CHM - WM | 87 | 4.77 | < .001 |
| CHM - NM | 87 | 8.20 | < .001 |
| WM - NM | 87 | 3.44 | < .001 |
| **Affective intention: high arousal, positive valence (H+)** |  |  |  |
| CHM - WM | 87 | 0.38 | .70 |
| CHM - NM | 87 | 1.86 | .20 |
| WM - NM | 87 | 1.48 | .29 |
| **Affective intention: low arousal, positive valence (L+)** |  |  |  |
| CHM - WM | 87 | –1.39 | .50 |
| CHM - NM | 87 | –1.26 | .50 |
| WM - NM | 87 | .13 | .90 |

Table S3b. Post hoc comparisons for listener groups across all instruments for the Measure context

| **Affective intention: low arousal, negative valence (L-)** | ***df*** | ***t* ratio** | ***p* value** |
| --- | --- | --- | --- |
| CHM - WM | 87 | 6.79 | < .001 |
| CHM - NM | 87 | 4.88 | < .001 |
| WM - NM | 87 | –1.91 | .06 |
| **Affective intention: high arousal, negative valence (H-)** |  |  |  |
| CHM - WM | 87 | 3.59 | < .001 |
| CHM - NM | 87 | 7.22 | < .001 |
| WM - NM | 87 | 3.64 | < .001 |
| **Affective intention: high arousal, positive valence (H+)** |  |  |  |
| CHM - WM | 87 | 5.22 | < .001 |
| CHM - NM | 87 | 5.13 | < .001 |
| WM - NM | 87 | –0.09 | .93 |
| **Affective intention: low arousal, positive valence (L+)** |  |  |  |
| CHM - WM | 87 | –0.24 | .83 |
| CHM - NM | 87 | 0.86 | .83 |
| WM - NM | 87 | 1.10 | .83 |

Table S3c. Post hoc comparisons for listener groups across all instruments for the Phrase context

| **Affective intention: low arousal, negative valence (L-)** | ***df*** | ***t* ratio** | ***p* value** |
| --- | --- | --- | --- |
| CHM - WM | 87 | 4.79 | < .001 |
| CHM - NM | 87 | 3.32 | .003 |
| WM - NM | 87 | –1.47 | .14 |
| **Affective intention: high arousal, negative valence (H-)** |  |  |  |
| CHM - WM | 87 | 4.27 | < .001 |
| CHM - NM | 87 | 5.99 | < .001 |
| WM - NM | 87 | 1.73 | .09 |
| **Affective intention: high arousal, positive valence (H+)** |  |  |  |
| CHM - WM | 87 | 2.98 | .007 |
| CHM - NM | 87 | 4.55 | < .001 |
| WM - NM | 87 | 1.56 | .12 |
| **Affective intention: low arousal, positive valence (L+)** |  |  |  |
| CHM - WM | 87 | –0.59 | .55 |
| CHM - NM | 87 | 1.41 | .32 |
| WM - NM | 87 | 2.01 | .14 |

Table S4. Post hoc comparisons for affective intentions across all listener groups and instruments for each context

| **Note** | ***df*** | ***t* ratio** | ***p* value** |
| --- | --- | --- | --- |
| (H-) - (H+) | 2001 | 14.04 | < .001 |
| (H-) - (L-) | 2001 | 6.64 | < .001 |
| (H-) - (L+) | 2001 | 20.49 | < .001 |
| (H+) - (L-) | 2001 | –7.41 | < .001 |
| (H+) - (L+) | 2001 | 6.45 | < .001 |
| (L-) - (L+) | 2001 | 13.86 | < .001 |
| **Measure** |  |  |  |
| (H-) - (H+) | 2001 | 5.97 | < .001 |
| (H-) - (L-) | 2001 | –0.90 | 0.37 |
| (H-) - (L+) | 2001 | 19.94 | < .001 |
| (H+) - (L-) | 2001 | –6.88 | < .001 |
| (H+) - (L+) | 2001 | 13.97 | < .001 |
| (L-) - (L+) | 2001 | 20.85 | < .001 |
| **Phrase** |  |  |  |
| (H-) - (H+) | 2001 | –6.26 | < .001 |
| (H-) - (L-) | 2001 | –0.44 | 0.66 |
| (H-) - (L+) | 2001 | 10.14 | < .001 |
| (H+) - (L-) | 2001 | 5.82 | < .001 |
| (H+) - (L+) | 2001 | 16.40 | < .001 |
| (L-) - (L+) | 2001 | 10.58 | < .001 |

Table S5a. Post hoc ANOVAs for listener group x instrument for the Note context

| **(L-) Valence** | **Df** | **Df residuals** | **F** | ***p*** | **signif.** |
| --- | --- | --- | --- | --- | --- |
| Listener Group | 2 |  | 12.51 | < .001 | *** |
| Instrument | 5 |  | 10.94 | < .001 | *** |
| Listener Group × Instrument | 10 | 522 | 6.73 | < .001 | *** |
| **(L-) Arousal** |  |  |  |  |  |
| Listener Group | 2 |  | 10.07 | < .001 | *** |
| Instrument | 5 |  | 96.19 | < .001 | *** |
| Listener Group × Instrument | 10 | 522 | 3.62 | < .001 | *** |
| **(H-) Valence** |  |  |  |  |  |
| Listener Group | 2 |  | 34.42 | < .001 | *** |
| Instrument | 5 |  | 13.14 | < .001 | *** |
| Listener Group × Instrument | 10 | 522 | 8.68 | < .001 | *** |
| **(H-) Arousal** |  |  |  |  |  |
| Listener Group | 2 |  | 96.19 | < .001 | *** |
| Instrument | 5 |  | 50.81 | < .001 | *** |
| Listener Group × Instrument | 10 | 522 | 1.03 | 0.42 |  |
| **(H+) Valence** |  |  |  |  |  |
| Listener Group | 2 |  | 3.59 | 0.03 | * |
| Instrument | 5 |  | 11.53 | < .001 | *** |
| Listener Group × Instrument | 10 | 522 | 4.59 | < .001 | *** |
|  |  |  |  |  |  |
| **(H+) Arousal** |  |  |  |  |  |
| Listener Group | 2 |  | 27.35 | < .001 | *** |
| Instrument | 5 |  | 38.68 | < .001 | *** |
| Listener Group × Instrument | 10 | 522 | 0.61 | 0.81 |  |
| **(L+) Valence** |  |  |  |  |  |
| Listener Group | 2 |  | 3.57 | 0.03 | * |
| Instrument | 5 |  | 13.35 | < .001 | *** |
| Listener Group × Instrument | 10 | 522 | 5.18 | < .001 | *** |
| **(L+) Arousal** |  |  |  |  |  |
| Listener Group | 2 |  | 10.02 | < .001 | *** |
| Instrument | 5 |  | 12.78 | < .001 | *** |
| Listener Group × Instrument | 10 | 522 | 3.08 | < .001 | *** |

Signif. codes: *** .001 **.01 *.05

Table S5b. Post hoc ANOVAs for listener group x instrument for the Measure context

| **(L-) Valence** | **Df** | **Df residuals** | **F** | ***p*** | **signif.** |
| --- | --- | --- | --- | --- | --- |
| Listener Group | 2 |  | 52.64 | < .001 | *** |
| Instrument | 5 |  | 18.28 | < .001 | *** |
| Listener Group × Instrument | 10 | 522 | 5.47 | < .001 | *** |
| **(L-) Arousal** |  |  |  |  |  |
| Listener Group | 2 |  | 92.57 | < .001 | *** |
| Instrument | 5 |  | 66.60 | < .001 | *** |
| Listener Group × Instrument | 10 | 522 | 3.23 | < .001 | *** |
| **(H-) Valence** |  |  |  |  |  |
| Listener Group | 2 |  | 52.87 | < .001 | *** |
| Instrument | 5 |  | 15.25 | < .001 | *** |
| Listener Group × Instrument | 10 | 522 | 11.65 | < .001 | *** |
| **(H-) Arousal** |  |  |  |  |  |
| Listener Group | 2 |  | 95.13 | < .001 | *** |
| Instrument | 5 |  | 32.25 | < .001 | *** |
| Listener Group × Instrument | 10 | 522 | 4.26 | < .001 | *** |
| **(H+) Valence** |  |  |  |  |  |
| Listener Group | 2 |  | 38.41 | < .001 | *** |
| Instrument | 5 |  | 21.02 | < .001 | *** |
| Listener Group × Instrument | 10 | 522 | 5.89 | < .001 | *** |
|  |  |  |  |  |  |
| **(H+) Arousal** |  |  |  |  |  |
| Listener Group | 2 |  | 35.12 | < .001 | *** |
| Instrument | 5 |  | 29.83 | < .001 | *** |
| Listener Group × Instrument | 10 | 522 | 1.47 | 0.15 |  |
| **(L+) Valence** |  |  |  |  |  |
| Listener Group | 2 |  | 19.64 | < .001 | *** |
| Instrument | 5 |  | 19.09 | < .001 | *** |
| Listener Group × Instrument | 10 | 522 | 4.52 | < .001 | *** |
| **(L+) Arousal** |  |  |  |  |  |
| Listener Group | 2 |  | 80.90 | < .001 | *** |
| Instrument | 5 |  | 32.66 | < .001 | *** |
| Listener Group × Instrument | 10 | 522 | 3.01 | 0.001 | ** |

Signif. codes: *** .001 **.01 *.05

Table S5c. Post hoc ANOVAs for listener group x instrument for the Phrase context

| **(L-) Valence** | **Df** | **Df residuals** | **F** | ***p*** | **signif.** |
| --- | --- | --- | --- | --- | --- |
| Listener Group | 2 |  | 12.25 | < .001 | *** |
| Instrument | 5 |  | 18.78 | < .001 | *** |
| Listener Group × Instrument | 10 | 522 | 3.19 | < .001 | *** |
| **(L-) Arousal** |  |  |  |  |  |
| Listener Group | 2 |  | 40.60 | < .001 | *** |
| Instrument | 5 |  | 42.92 | < .001 | *** |
| Listener Group × Instrument | 10 | 522 | 1.55 | 0.12 |  |
| **(H-) Valence** |  |  |  |  |  |
| Listener Group | 2 |  | 36.93 | < .001 | *** |
| Instrument | 5 |  | 16.76 | < .001 | *** |
| Listener Group × Instrument | 10 | 522 | 8.44 | < .001 | *** |
| **(H-) Arousal** |  |  |  |  |  |
| Listener Group | 2 |  | 50.76 | < .001 | *** |
| Instrument | 5 |  | 13.59 | < .001 | *** |
| Listener Group × Instrument | 10 | 522 | 4.32 | < .001 | *** |
| **(H+) Valence** |  |  |  |  |  |
| Listener Group | 2 |  | 28.94 | < .001 | *** |
| Instrument | 5 |  | 16.12 | < .001 | *** |
| Listener Group × Instrument | 10 | 522 | 3.35 | < .001 | *** |
|  |  |  |  |  |  |
| **(H+) Arousal** |  |  |  |  |  |
| Listener Group | 2 |  | 16.57 | < .001 | *** |
| Instrument | 5 |  | 18.50 | < .001 | *** |
| Listener Group × Instrument | 10 | 522 | 1.23 | 0.27 |  |
| **(L+) Valence** |  |  |  |  |  |
| Listener Group | 2 |  | 8.33 | < .001 | *** |
| Instrument | 5 |  | 21.20 | < .001 | *** |
| Listener Group × Instrument | 10 | 522 | 5.05 | < .001 | *** |
| **(L+) Arousal** |  |  |  |  |  |
| Listener Group | 2 |  | 40.29 | < .001 | *** |
| Instrument | 5 |  | 30.68 | < .001 | *** |
| Listener Group × Instrument | 10 | 522 | 2.93 | 0.001 | ** |

Signif. codes: *** .001 **.01 *.05
